# Asymmetric interactions between two butterfly species mediated by food demand

**DOI:** 10.1002/ece3.10164

**Published:** 2023-06-08

**Authors:** Koya Hashimoto, Takayuki Ohgushi

**Affiliations:** ^1^ Center for Ecological Research Kyoto University Otsu Japan; ^2^ Present address: Department of Biology, Faculty of Agriculture and Life Science Hirosaki University Hirosaki Japan

**Keywords:** aristolochic acids, *Atrophaneura alcinous*, biomass‐mediated indirect interaction, herbivorous insects, interaction symmetry, *Sericinus montela*

## Abstract

Recent studies on insect interactions on plants have revealed that herbivorous insects indirectly interact with each other through changes in plant traits following herbivory. However, less attention has been given to plant biomass relative to plant quality in relation to indirect interactions among herbivores. We explored the extent to which the larval food demand of two specialist butterflies (*Sericinus montela* and *Atrophaneura alcinous*) explains their interaction on a host plant, *Aristolochia debilis*. A laboratory experiment showed that plant mass consumption by *A. alcinous* larvae was 2.6 times greater than that by *S. montela*. We predicted that *A. alcinous*, which requires more food, is more vulnerable to food shortages than *S. montela*. In a cage experiment, an asymmetric interspecific interaction was detected between the two specialist butterflies; *S. montela* larval density significantly decreased the survival and prolonged the development time of *A. alcinous*, but *A. alcinous* density affected neither the survival nor the development time of *S. montela*. The prediction based on the food requirement was partly supported by the fact that increasing *A. alcinous* density likely caused a food shortage, which more negatively affected *A. alcinous* survival than *S. montela* survival. Conversely, increasing the density of *S. montela* did not reduce the remaining food quantity, suggesting that the negative effect of *S. montela* density on *A. alcinous* was unlikely to be due to food shortage. Although aristolochic acid I, a defensive chemical specific to *Aristolochia* plants, did not influence the food consumption or growth of either butterfly larva, unmeasured attributes of plant quality may have mediated an indirect interaction between the two butterflies. Consequently, our study suggests that not only the quality but also the quantity of plants should be considered to fully understand the characteristics, such as symmetry, of interspecific interactions among herbivorous insects on the same host plant.

## Graphical abstract

Although previous studies suggest that asymmetry in plant‐mediated indirect interactions between herbivorous insects is mediated by changes in plant quality (i.e., defensive chemicals and/or nutrition), the roles of plant quantity in determining the symmetry/asymmetry of interactions between herbivores have been poorly explored. Our study shows an asymmetric interaction between two specialist butterfly species; the species with greater food demand experienced negative effects from the other, but the reverse did not occur. We found that a reduction in plant biomass less negatively affected the larval performance of the species with lower food demand, generating asymmetry in the interspecific interactions.

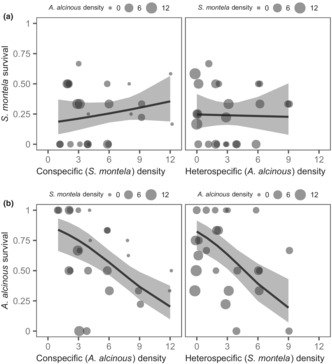

## 1 INTRODUCTION

It is widely accepted that herbivorous insects indeed interact with one another via their shared host plants in various ways (Denno & Kaplan, 2007; Ohgushi, 2005), although mechanistic understanding of such indirect interactions among herbivores, such as the relative contributions of different mechanisms, is still limited (Anderson et al., 2009; Kaplan & Denno, 2007). There are at least two potential factors generating plant‐mediated interspecific interactions between herbivorous insects: plant quantity (biomass) and quality (i.e., secondary substances or nutrition conditions). On the one hand, exploitative competition can occur when plant biomass reduced by herbivory decreases food availability for the other herbivore species. On the other hand, insect attacks alter plant quality, such as secondary chemicals and nutrients (Karban & Baldwin, 1997), which affects the survival and/or reproduction of subsequently colonizing herbivores (Viswanathan et al., 2005, 2007). In a broader context, the former and the latter can be classified as density‐ and trait‐mediated indirect interactions, respectively (Abrams, 1995), both of which are considered structuring forces of ecological communities (van Veen et al., 2006).

Although both factors can generate indirect interactions between herbivorous insects, to date, researchers have paid more attention to plant quality than to plant quantity. This is partly because there is a long‐standing idea that host plants are rarely depleted by insect feeding, and thus, exploitative competition between herbivorous insects is unlikely to occur (Lawton & Strong, 1981; Strong et al., 1984). In reality, however, food depletion caused by herbivorous insects occurs in nature. Population dynamics research on herbivorous insects has shown the negative effects of food shortages, especially for species feeding on herbaceous plants (Dempster, 1983; van der Meijden, 1979). For example, outbreaks of the cinnabar moth often cause defoliation of its food plant ragwort, resulting in high larval mortality due to starvation (Dempster, 1971, 1983). Such negative effects of food shortages on herbivore performance due to their consumption lead to negative density dependence of their population growth rate (Dempster, 1971). Additionally, food shortages can result in local extinction and/or emigration of herbivorous insects, thereby driving metapopulation dynamics (Hanski, 1999; Harrison et al., 2011; van der Meijden, 1979). Given these effects of food shortage by herbivore consumption, reduced plant biomass can negatively affect not only conspecifics but also heterospecifics, and interspecific interactions among herbivorous insects should be regarded as a mixture of plant quality‐ and quantity‐mediated interactions (Anderson et al., 2009). In other systems, such as prey–predator food webs, an integrated understanding of complex density‐ and trait‐mediated interactions has been growing, such as their relative importance and context dependency (Hoverman & Relyea, 2012; Preisser et al., 2005; Pruett & Weissburg, 2021; Takagi & Miyashita, 2015; Wada et al., 2017; but see Okuyama & Bolker, 2007, 2012). However, although some studies have shown the occurrence of plant biomass‐mediated interspecific interactions between herbivorous insects (Branson, 2010; Branson & Haferkamp, 2014; Crawley & Pattrasudhi, 1988; Hudson & Stiling, 1997; Morris, 1997), little is known about the extent to which such plant biomass‐mediated effects are reflected in the characteristics of herbivore interactions observed in nature.

One approach to understanding plant quantity‐mediated interactions between herbivorous insects is to examine whether herbivore traits related to food resource utilization affect interaction symmetry. Although traditional ecology generally assumes that symmetric exploitative competition is the only interaction among consumers mediated by their shared food resources (Lawton & Hassell, 1981; Strong et al., 1984), recent reviews have argued that asymmetric interactions among herbivorous insects are common and that plant quality is responsible for those interactions (Denno & Kaplan, 2007; Kaplan & Denno, 2007). However, it is unclear whether plant quantity, not quality, accounts for the asymmetry in plant‐mediated interactions between herbivores. In this context, the food demand of an herbivore species to fulfill its development would be an important factor accounting for the symmetry/asymmetry of interactions mediated by plant quantity. In theory, a consumer with greater resource demand should be an inferior competitor because its survival and/or development is more likely lowered by resource shortages (Chesson, 2000; Tilman, 1982). If this is applicable to herbivorous insects, food demand to fulfill development would influence interspecific interactions. Specifically, species with greater food demand are more likely to be affected by food shortages by con‐ and heterospecifics than species with lower food demand. As such, an asymmetric interaction, in which the species with greater food demand is more negatively affected by another species with lower food demand, likely occurs. Testing this prediction may provide insight into the contribution of plant quantity to herbivore–herbivore interactions.

*Sericinus montela* Gray and *Atrophaneura alcinous* (Klug) are specialist papilionid butterflies that utilize a common host plant, *Aristolochia debilis* Sieb. et Zucc., which provides a suitable study system to explore indirect interactions between herbivorous insects mediated by plant biomass. *Aristolochia debilis* is reportedly often depleted by the feeding of these butterfly larvae in the field (Inoue & Kon, 2006). *Sericinus montela* is an exotic species in Japan, while *A. alcinous* is a native species, and they have co‐occurred for more than 25 years in a study area in Kyoto Prefecture (Hashimoto & Ohgushi, 2017). As *A. debilis* contains toxic aristolochic acids, which are putative defensive chemicals, the plants are rarely attacked by other herbivores. Moreover, these butterflies sequester aristolochic acids to utilize for their own defense against natural enemies (Nishida, 1994; Nishida & Fukami, 1989). This defensive substance may increase feeding by these herbivores and decrease the abundance of *A. debilis* (cf. Carson & Root, 1999). Additionally, as herbivory induces aristolochic acid production in other *Aristolochia* species (Fordyce, 2001), a negative interaction mediated by plant quality may occur between these butterflies. Our previous study showed that *A. debilis* leaves regrown after herbivory by *S. montela* and/or *A. alcinous* did not decrease or increase the larval growth of either species (Hashimoto & Ohgushi, 2017). Given that herbivore‐induced plant responses often depend on the time elapsed after herbivory (Underwood, 1998), however, it is possible that previous herbivory on *A. debilis* affects subsequent herbivores before the damaged plants regrow (i.e., a shorter time scale than in the previous study). Note that specialist herbivores are generally less affected by plant defensive chemicals, depending on herbivore species identity (Ali & Agrawal, 2012; Cornell & Hawkins, 2003). It is not known whether the larval performance of these two butterfly species is affected by aristolochic acids.

The aim of this study was to explore whether the food demand of these butterfly larvae generates plant‐mediated interactions between *S. montela* and *A. alcinous*. Specifically, we ask the following questions: (1) Which species has greater food demand per individual larva? (2) To what extent do the food demands of these butterflies explain the effects of conspecific and heterospecific density on the survival and development time of butterfly larvae? (3) Does the addition of aristolochic acid affect larval consumption or growth?

## 2 MATERIALS AND METHODS

### 2.1 Study organisms

*Sericinus montela* (Papilionidae, Parnassiini) is an exotic butterfly species in Japan that was originally distributed across Korea, China, and the Maritime Province in Russia. It was introduced to Tokyo Prefecture in the late 1970s and expanded its distribution to central and western Japan thereafter (Matsuka & Ohno, 1981; Nakamura, 2010; Sakuratani et al., 2003). *Atrophaneura alcinous* (Papilionidae, Troidini) is a native butterfly species in Japan. These two butterfly species are multivoltine and have similar life histories. Adults of the first generation emerge in early spring, and both species produce several generations a year and overwinter in the pupal stage. In Kyoto Prefecture, where *S. montela* was first recorded in 1993 (Shoji, 1997), the host plant of *S. montela* is *A. debilis*, which is shared with the native butterfly species *A. alcinous*. *Aristolochia debilis* is a perennial, herbaceous vine. The aboveground plant parts wither in November–March, and new shoots emerge from overwintering roots in early spring.

### 2.2 Estimation of the food amount required for larval growth

To investigate the food demand of larvae of the two species, we evaluated the mass of leaves consumed over the period from hatching until pupation in the laboratory. The experiment was conducted from June 11 to July 21, 2016. Eggs obtained from plants growing on riverbanks of the Kizu River in June 2016 were kept in an environmental chamber at 23°C and 14 L:10D until hatching. Ten neonate larvae were placed in a plastic case (7.5 cm × 9 cm × 4 cm) with fresh leaves of *A. debilis* grown in a common garden at the Center for Ecological Research, Kyoto University (Shiga Prefecture), and were kept in a chamber (23°C, 14 L:10D). The leaves for the experiment were taken from the sixth to ninth nodes from the top of growing shoots of *A. debilis*. When more than half of the larvae molted into the 3rd instar, we placed one larva in a plastic case (7.5 cm × 9 cm × 4 cm). The timing of molting into the 3rd instar of all experimental larvae occurred in less than 2 days in each species. Ample amounts of fresh leaves were provided every 2 days, and we measured the fresh masses of larvae, newly provided leaves, and remaining leaves. In addition to this experiment, we collected additional larvae to determine the relationship between the fresh mass and dry mass of larvae (*S. montela*: ln(dry mass) = −1.66 + 1.08 × ln(fresh mass), *R* ^2^ = .99; *A. alcinous*: ln(dry mass) = −1.98 + 1.05 × ln(fresh mass), *R* ^2^ = .99). By using these relationships, we converted the fresh mass of larvae to dry mass. Likewise, we collected additional *A. debilis* leaves to obtain the relationship between leaf fresh mass and dry mass and converted fresh mass to dry mass (ln(dry mass) = −1.48 + 0.90 × ln(fresh mass), *R* ^2^ = .97). Larval growth was obtained from the cumulative gain of dry mass, and leaf consumption was the total dry mass of leaves consumed, which was calculated by subtracting the dry mass of the remaining leaves from that of the leaves provided 2 days before.

### 2.3 Con‐ and heterospecific density effects on the survival and development time of larvae

**FIGURE 1:**
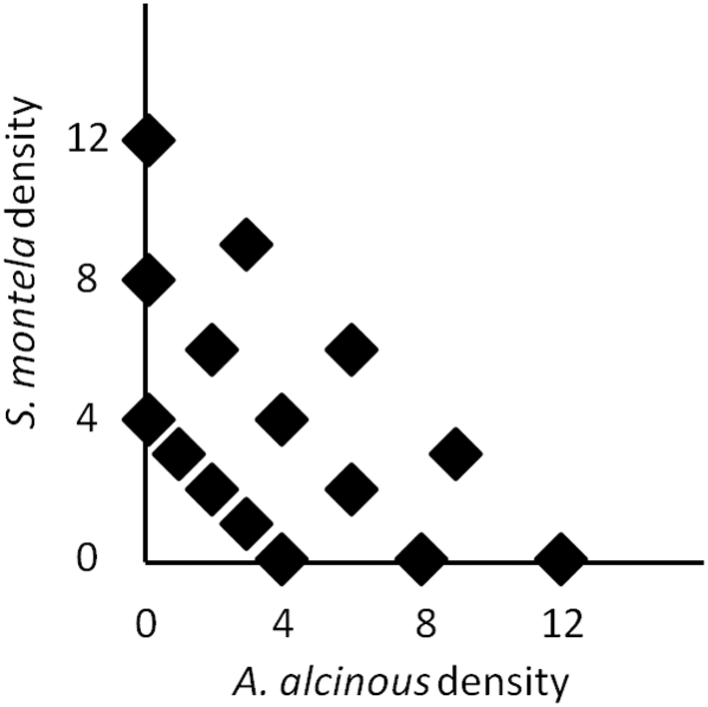
Experimental design used to evaluate con‐ and heterospecific density effects on larval performance (i.e., survival and development time).

To examine whether con‐ and heterospecific larval density affects larval survival and development time, we conducted a common garden experiment to manipulate larval numbers of *S. montela* and *A. alcinous* independently. Although this design did not permit many replicates of the respective treatments, it allowed us to evaluate how con‐ and heterospecific density effects differ from each other (Inouye, 2001). The total density (the sum of *S. montela* and *A. alcinous* individuals) was 4, 8, and 12 individuals, and the ratio of *S. montela* to *A. alcinous* was set at 0:1 (*S. montela* alone), 1:3, 1:1, 3:1 (mixed species), and 1:0 (*A. alcinous* alone) for each of the three total density values mentioned above (Figure 1). This design had a total of 15 combinations of *S. montela* and *A. alcinous* larvae with two replicates of each combination. Our field survey at the Kizu River from 2013 to 2016 showed that the numbers of *S. montela* and *A. alcinous* larvae (1st–3rd instar) per plant were 13.6 ± 16.8 and 3.1 ± 3.0 (mean ± SD), respectively. Thus, the range of larval numbers used in the experiment was similar to that observed in the field, as was the species ratio. Note that our design did not include manipulation of plant quantity but did include manipulation of larval density because the specific objective of our experiment was not to test the occurrence of plant quantity‐mediated herbivore interactions but to explore the extent to which the observed patterns of interactions between the herbivores matched the predictions from the theory of resource density‐mediated interactions.

The field experiment was conducted in September–October 2012 at a common garden of the Center for Ecological Research. Thirty cages (0.9 m × 0.9 m × 1 m) made of 1‐mm polyethylene net mesh and weed barrier mat were placed in 10 greenhouses (1.8 m × 1.8 m × 2 m) at the garden. Each greenhouse had three cages. The north and south sides of the vinyl sheets that covered the greenhouses were removed for ventilation. The upper part of each greenhouse was covered by sunshade net. Plants were propagated from roots collected from the bank of the Kizu River, Kyoto Prefecture, in spring 2011, planted in plastic pots (12 cm in diameter) with compost, and grown in a greenhouse. Plant roots were transplanted to new pots in spring 2012. Newly hatched larvae were used for the experiment. Eggs were obtained from adult females collected at the Kizu River in September 2011. The eggs were kept in a growth chamber (23°C, 14 L:10D) until they hatched.

The 15 treatments (Figure 1) with two replicates each were randomly assigned to each cage. Prior to the experiment, the number of leaves of each plant was counted, and four potted plants were placed in each cage (see Figure S1 for the spatial arrangement in a cage). The mean number of leaves per cage was 348.9 (SD = 7.5). There were no significant correlations between the number of leaves and assigned *S. montela* or *A. alcinous* density (*S. montela* density: Pearson's *r* = −.22, *t*_28_ = −1.21, *p* = .24; *A. alcinous* density: Pearson's *r* = −.09, *t*_28_ = −0.50, *p* = .62), confirming that larval density effects would not be confounded by the effects of leaf abundance. We placed newly hatched *S. montela* and *A. alcinous* larvae in each assigned treatment in each cage (Figure 1). The larvae of *S. montela* and *A. alcinous* were separately placed on different potted plants in each cage (see Figure S1). This design corresponded to the spatial distribution in the field, where *S. montela* and *A. alcinous* neonates are unlikely to occur together on individual plants (personal observation by the first author). Additionally, larvae of these butterflies often move from the original plants on which they hatched to other plants before pupation. Hence, as they grew, the two species may have interacted with each other on the same plants through interplant movements. In fact, we observed the situation where both species occurred on the same plant simultaneously, but not frequently. After the experiment was established, the number of larvae and their developmental stage were recorded every 2 days. This survey lasted until all larvae died or pupated (7 weeks after the experiment started), except for one cage in which *S. montela* larvae could not complete their development, probably due to low temperatures. The date when each pupa was first recorded was used to estimate the development time of each larva from hatching to pupation. We compared the larval survival in each cage (number of larvae pupated/initial number of larvae) and development time of individual larvae (days from hatching until pupation) among the treatments. At the end of the experiment, we visually classified the percentage of final food remaining (100−percentage of leaf consumption) into five categories: 0% (no leaves or stems left), <25%, 25%–50%, 50%–75%, and >75% of aboveground plant tissue.

### 2.4 Effects of the addition of aristolochic acid I on the leaf consumption and growth of larvae

To test whether the addition of aristolochic acid I (AAI) affects the performance of *S. montela* and *A. alcinous* larvae, we conducted a laboratory experiment. There are some other aristolochic acids present in *Aristolochia* species other than AAI (e.g., AAII and AAIII; Nishida & Fukami, 1989), but it has been reported that AAI is the most abundant and toxic compound (Balachandran et al., 2005; Lajide et al., 1993; Nishida & Fukami, 1989). We reared *S. montela* and *A. alcinous* larvae by providing *A. debilis* leaves with and without AAI application. The leaves used in the experiment were randomly collected from the stock of *A. debilis* in the common garden. One leaf was cut in half from its central vein, and each side of the leaf was randomly assigned to the AAI application treatment or control. The addition of AAI was performed similarly to that described in Dimarco et al. (2012) and Dimarco and Fordyce (2017). We applied approximately 30 μg of AAI (in 200 μg AAI/1 mL 99% ethanol) to half of the leaves using a paint brush. Our preliminary survey showed that an *A. debilis* leaf contained 25.53 ± 18.19 μg (mean ± SD) of AAI. Thus, it is expected that half of the leaves treated with AAI contained approximately three times more AAI than observed under natural conditions. We also applied 99% ethanol to the other half (i.e., control) in the same manner. Although we did not compare the concentration of AAI between AAI‐treated and control leaves, Dimarco and Fordyce (2017) confirmed that the addition of AAI by 99% ethanol solution successfully elevated the AAI content in *Aristolochia* leaves. Each treated leaf was provided to one third instar larva of either *S. montela* or *A. alcinous* (*S. montela*: *n* = 60; *A. alcinous*: *n* = 39). The larvae were allowed to feed on the leaves for 24 h. Before and after the experiment, we measured the area of each half leaf and the fresh weight of the larvae to obtain the consumed leaf area. The consumed leaf area was estimated by subtracting the unconsumed leaf area from the initial leaf area. We compared the relative growth rate (RGR) and relative consumption rate (RCR) of the larvae between food treatments with and without AAI application. We calculated RGR and RCR as follows:

$$\mathrm{RGR}=\left(\text{final larval weight}-\text{initial larval weight}\right)/\text{initial larval weight}$$

RCR=consumed leaf area/initial larval weight

### 2.5 Statistical analysis

To describe the inter‐plant movement of both butterfly larvae, we analyzed snapshot data of the spatial distribution of the two butterfly larvae in each cage (day = 1, 7, 15, 21, 29, and 42 (almost the end of the experiment)). On each day, we examined intraspecific spatial aggregation and interspecific spatial segregation/overlap among the four plants in each cage. Specifically, intraspecific aggregation was expressed by Morisita's *I*_*δ*_ index (Morisita, 1959). When 0 ≤ *I*_*δ*_ < 1, *I*_*δ*_ = 1 and 1 < *I*_*δ*_ < +∞, the distribution of larvae is more dispersed, random, and more aggregated, respectively. Interspecific segregation/overlap was given by Iwao's *ω* index (Iwao, 1977). When −1 ≤ *ω* < 0, *ω* = 0, and 0 < *ω* ≤ 1, the interspecific distribution is more segregated, random, and more overlapping, respectively. Both indices are independent of density, which is appropriate for our experiment in which larval density decreased as time elapsed. Based on these snapshot distribution data, we counted the number of plants in each cage that were visited at least once by either of these butterfly species each day.

The effects of larval density on larval survival were analyzed separately for the two species by generalized linear models (GLMs) with a binomial error structure and a logit link function. The models contained the density of each species (continuous variable) and their interaction as explanatory variables and the probability of larval survival as a response variable. Because the *S. montela* and *A. alcinous* larval numbers were independent in our experiment, we could separate con‐ and heterospecific density effects. We tested the significance of each regression coefficient by Wald *z* tests. When the interaction terms were not significant, we reported the results of models without interactions.

The effects of larval density on larval development time (days from egg hatching to pupation) were analyzed by linear mixed models with the “lme4” package version 1.1.25 (Bates et al., 2015). Data for development times were log‐transformed to meet the model assumptions of normality and homoscedasticity. The models contained the density of each species and their interactions as explanatory variables and cage as a random effect. Development time was a response variable. We tested the significance of each regression coefficient by Wald *t*‐tests with Satterthwaite approximate degrees of freedom computed by the “lmerTest” package version 3.1.3 (Kuznetsova et al., 2017). For the survival analysis, we removed interaction terms from the models when they were not significant.

We analyzed the effects of initial *S. montela* and *A. alcinous* density on the final food remaining using a cumulative logit model with the “ordinal” package version 2019.12.10 (Christensen, 2019). The model contained the density of each species and their interaction as explanatory variables and cage as a random effect. Final food remaining (ordinal variable with five categories: 0% (no leaves or stems left), <25%, 25–50%, 50–75%, and > 75% of aboveground plant tissue) was included as a response variable. The significance of the regression coefficients was tested by Wald *z* tests as described above. Regarding all the above analyses, we transformed all the explanatory variables to have a mean of zero by subtracting the mean value from the original variable values (i.e., “centering”) and then computed statistics such as partial regression coefficients and *t* values to facilitate interpretation of the results. Specifically, when centering explanatory variables, the effects of each explanatory variable can be interpreted as the effects when the other variables are set to their original mean values (Schielzeth, 2010).

The effects of AAI application on leaf consumption and larval growth were analyzed by linear mixed models. The models contained AAI application (addition or no addition) as an explanatory variable and larval RCR and RGR as response variables. The experimental leaves were included as a random effect. The significance of the explanatory variable was tested by Wald *t*‐tests using Satterthwaite approximate degrees of freedom as in the above analyses.

All statistical analyses were conducted using R statistical software version 3.6.3 (R Core Team, 2020).

## 3 RESULTS

### 3.1 Food demand for larval development

*Atrophaneura alcinous* had greater leaf consumption and larval growth than *S. montela* (Table 1). In fact, *A. alcinous* larvae required 2.6 times more plant mass for feeding to fulfill larval development than *S. montela* larvae.

**TABLE 1:** Growth and leaf consumption of *Sericinus montela* and *Atrophaneura alcinous* larvae.

	Stage	Species	
		*Sericinus montela*	*Atrophaneura alcinous*
Larval growth (mg dry weight)	1st–2nd instar	0.49	5.43
	3rd–5th instar	48.33 (±1.21)	151.33 (±4.22)
	Total	48.82	156.76
Leaf consumption (mg dry weight)	1st–2nd instar	1.88	21.66
	3rd–5th instar	394.25 (±9.10)	1003.23 (±39.56)
	Total	396.13	1024.89

*Note*: Mean ± SE are presented for the 3rd–5th instars. Mean values without SE are presented for the 1st–2nd instars because we reared 10 1st–2nd instar larvae together in one plastic case.

### 3.2 Temporal changes in the numbers and spatial patterns of the larvae in the cage experiment

Total numbers of *S. montela* and *A. alcinous* larvae decreased due to death or pupation as time elapsed (Figure S2a,b). Six weeks after the experiment started (1 week before the experiment was terminated), almost all larvae died or pupated (Figure S2a,b). As time elapsed, intraspecific spatial aggregation decreased in both species (Figure S2c,d), while interspecific overlapping increased (Figure S2e). This suggests that both species dispersed from the plants where they were initially placed and that the likelihood of encounter would have increased. The number of plants that had been visited by either of the two species also increased as time passed (Table S1). Six weeks after the experiment started, in 20/30 cages, all (four) plants were visited at least once by either of the two butterflies (Table S1c). Moreover, after 6 weeks, in cages with a higher initial density (total density = 8 and 12), almost all plants were once visited by either of the species, indicating that both butterfly larvae indeed frequently moved among plants and ate plants on which they were not initially placed.

### 3.3 Effects of larval density on larval survival and development

**FIGURE 2:**
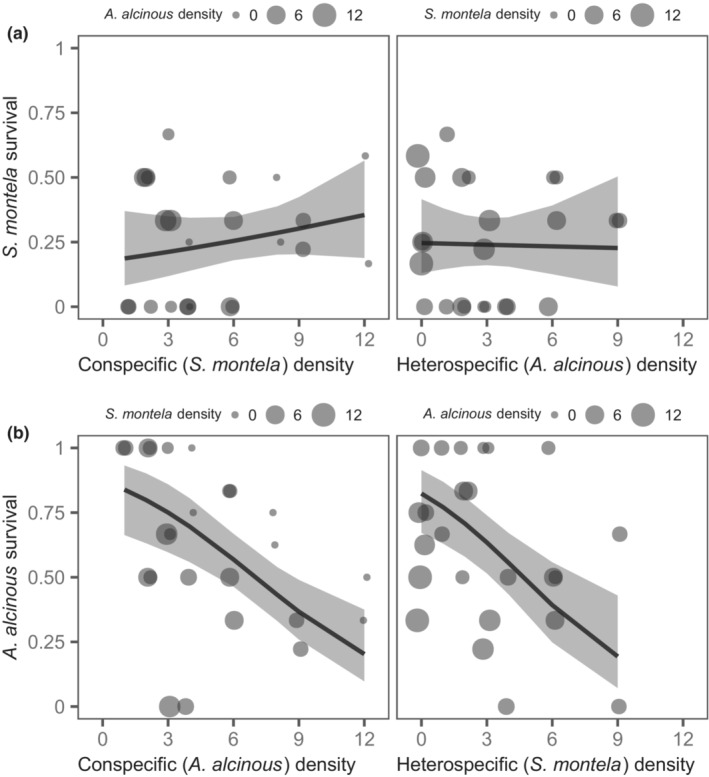
Effects of con‐ and heterospecific density on *Sericinus montela* larval survival (a) and *Atrophaneura alcinous* larval survival (b). Fitted curves with 95% confidence intervals were obtained with all covariates held at mean values. The *x*‐axis range of the curve is different between the left panel (1–12) and right panel (0–9) because there are no data points outside of the range, which is due to the treatment combinations in the experiment.

For larval survival of both *S. montela* and *A. alcinous*, the interaction between con‐ and heterospecific density was not significant (*S. montela* survival; *z* = −1.23, *p* = .22, *A. alcinous* survival; *z* = −0.39, *p* = .69), so we removed these interaction terms from the analyses. While larval survival of *S. montela* was not affected by con‐ or heterospecific density (Table 2a, Figure 2a), *A. alcinous* survival decreased with increasing con‐ and heterospecific density (Table 2a, Figure 2b). The partial regression coefficients of conspecific (*A. alcinous*) and heterospecific (*S. montela*) density did not significantly differ from each other (i.e., comparison between the coefficients of *A. alcinous* and *S. montela*, *χ* ^2^_1_ = 0.49, *p* = .48), suggesting that there was no significant difference in the effects of con‐ and heterospecific density.

**TABLE 2:** Effects of con‐ and heterospecific density on *Sericinus montela* and *Atrophaneura alcinous*. (a) Larval survival, (b) development time.

Butterfly species	Density	Coefficient	SE	*z*	*p*
(a)					
*Sericinus montela* survival	Conspecific density	0.079	0.073	1.08	.28
	Heterospecific density	−0.012	0.098	−0.12	.90
*Atrophaneura alcinous* survival	Conspecific density	−0.27	0.076	−3.60	**<.001**
	Heterospecific density	−0.33	0.097	−3.41	**<.001**

Butterfly species	Density	Coefficient	SE	df	*t*	*p*
(b)						
*Sericinus montela* development time	Conspecific density	−0.0051	0.012	13.81	0.44	.67
	Heterospecific density	0.013	0.015	20.83	0.89	.39
*Atrophaneura alcinous* development time	Conspecific density	0.014	0.0084	16.60	1.66	.12
	Heterospecific density	0.083	0.014	16.66	6.12	**<.001**
	Conspecific density × Heterospecific density	0.016	0.0041	15.66	3.87	**<.01**

*Note*: Bold shows statistical significance (α = 0.05).

**FIGURE 3:**
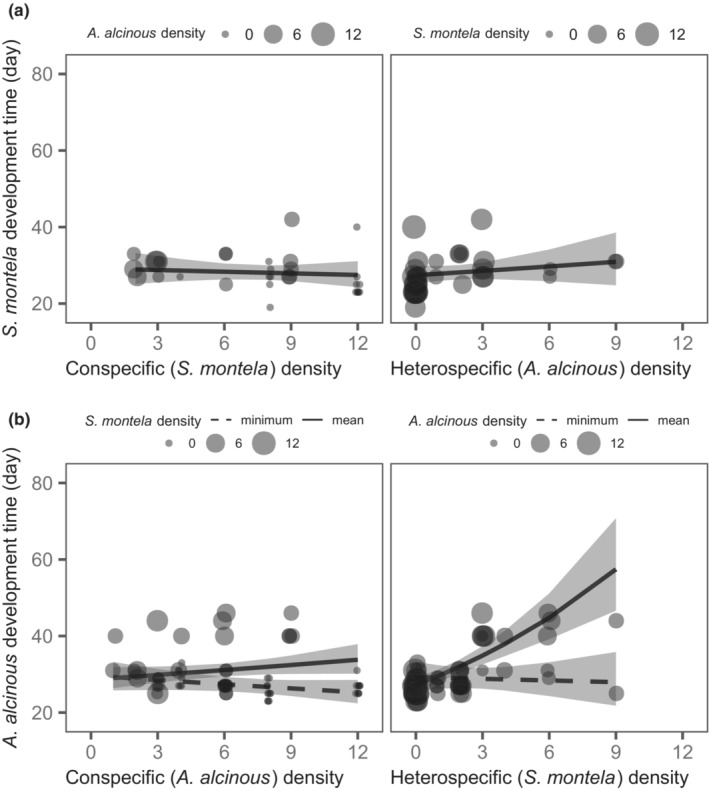
Effects of con‐ and heterospecific density on the larval development time of *Sericinus montela* (a) and *Atrophaneura alcinous* (b). Fitted curves with 95% confidence intervals are shown. In (a), fitted curves and confidence intervals were obtained with all covariates held at mean values. As the interaction term was significant for *A. alcinous* (see Table 2b), two fitted curves, in which *S. montela* density (left panel) and *A. alcinous* density (right panel) were the minimum (dashed curves) and mean (solid curves), are shown in (b). In the left panel, the solid curve and dashed curve were obtained when the *S. montela* density was held at the mean and minimum values, respectively. In the right panel, the solid curve and dashed curve were obtained when *A. alcinous* density was held at mean and minimum values, respectively. The *x*‐axis range of the curve is different among panels ([a] left: 2–12, [a] right: 0–9, [b] left: 1–12, [b] right: 0–9) because there are no data points outside of the range either due to the treatment combinations in the experiment or to a lack of survivors.

For the development time of *S. montela*, the conspecific × heterospecific density interaction was not significant (*t*_7.87_ = 1.71, *p* = .13). After removing this interaction, the development time of *S. montela* larvae was unaffected by *S. montela* and *A. alcinous* density (Table 2b, Figure 3a). In contrast, the development time of *A. alcinous* was dependent on the conspecific × heterospecific density interaction effect (*t*_15.66_ = 3.87, *p* < .05, Table 2b). Specifically, heterospecific (*S. montela*) density did not alter *A. alcinous* development time when other *A. alcinous* individuals were absent (i.e., *A. alcinous* density was at a minimum (=1)), but it significantly prolonged *A. alcinous* development time when *A. alcinous* density increased (Figure 3b). Higher *A. alcinous* density did not strongly affect *A. alcinous* development time (Figure 3b).

### 3.4 Effects of larval density on food consumption

**FIGURE 4:**
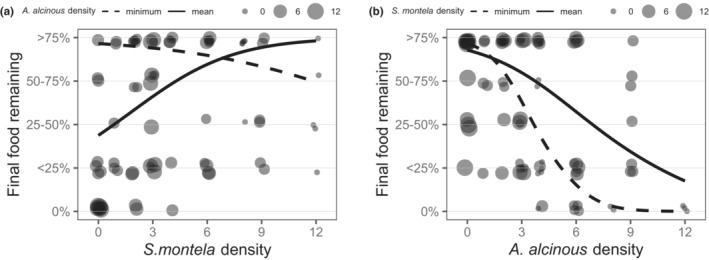
Effects of *Sericinus montela* (a) and *Atrophaneura alcinous* (b) density on final food remaining. Fitted curves are shown. Since the interaction term was significant (Table 3), fitted curves, in which the heterospecific densities were the minimum (dashed curves) and mean (solid curves), are shown. In panel (a), the solid curve and dashed curve were obtained when *A. alcinous* density was held at the mean and minimum values, respectively. In panel (b), the solid curve and dashed curve were obtained when the *S. montela* density was held at the mean and minimum values, respectively.

We detected a significant interaction effect between *S. montela* and *A. alcinous* density on final food remaining (Table 3). Both species decreased the remaining food amount when the heterospecific density was minimal (i.e., heterospecific density = 0), with a larger effect of *A. alcinous* than *S. montela* (Figure 4a,b). In particular, when the *S. montela* density was minimal (i.e., *S. montela* were absent), larvae of *A. alcinous* caused complete food depletion for all replicates when their initial densities were 8 and 12. However, when the density of heterospecifics increased, the negative density effects on food amount were ameliorated. Although the negative effect of *A. alcinous* density was still large when *S. montela* was present (Figure 4b), the effect of *S. montela* density changed from negative to positive when *A. alcinous* density increased (Figure 4a).

**TABLE 3:** Effects of *Sericinus montela* and *Atrophaneura alcinous* density on final food remaining.

Food status	Density	Coefficient	SE	*z*	*p*
Final food remaining	*Sericinus montela* density	0.39	0.13	2.97	**<.01**
	*Atrophaneura alcinous* density	−0.42	0.13	−3.24	**<.01**
	*Sericinus montela* density × *Atrophaneura alcinous* density	0.15	0.04	3.51	**<.001**

*Note*: Bold shows statistical significance (α = 0.05).

### 3.5 Effects of the addition of AAI on larval consumption and growth

Aristolochic acid application affected neither the leaf consumption nor the growth of either butterfly species. The RCR did not differ between the AAI treatment and control for *S. montela* and *A. alcinous* (*S. montela*; *t*_29.0_ = −0.39, *p* = .7, *A. alcinous*; *t*_16.9_ = 0.97, *p* = .4). Additionally, the RGR did not differ between the AAI treatment and control for *S. montela* and *A. alcinous* (*S. montela*; *t*_29.0_ = −0.018, *p* = 1.0, *A. alcinous*; *t*_16.2_ = 1.02, *p* = .3).

## 4 DISCUSSION

Our study clearly demonstrated the asymmetric interaction between the two specialist butterflies: *S. montela* larvae can negatively affect *A. alcinous* survival and development, but the reverse is unlikely to occur. We predicted that *A. alcinous* is more vulnerable to food shortages than *S. montela*. This is because the former has greater food demand than the latter (Table 1). Supporting this prediction, in the cage experiment, increasing *A. alcinous* density dramatically reduced the final food remaining (Figure 4b), which was accompanied by a significant decrease in *A. alcinous* survival (Figure 2b), but did not affect *S. montela* survival (Figure 2a). Although *S. montela* density significantly decreased *A. alcinous* survival (Figure 2b) and prolonged its development time (Figure 3b), the effect of consumption by *S. montela* was weak (Figure 4a). Additionally, an increase in *S. montela* density even increased the remaining food when grown with *A. alcinous* (Figure 4a, *A. alcinous* density = mean). This positive relationship between *S. montela* density and remaining food may be because the presence of *S. montela* suppressed the consumption effects of *A. alcinous* by decreasing *A. alcinous* survival. This suggests that the negative effect of *S. montela* density on *A. alcinous* was unlikely to have occurred due to direct food shortage. Furthermore, the addition of AAI did not influence food consumption and growth, suggesting that AAI is unlikely to contribute to an indirect interaction between the two butterflies. Taken together, our findings show that even in systems where food depletion frequently occurs, factors other than food shortage may mediate interspecific interactions among herbivorous insects.

### 4.1 Effects of species‐specific food demand on larval vulnerability to food shortage

In our study, food shortage (i.e., when the food amount available is below the threshold required for the development of a forager) more likely occurred due to feeding by *A. alcinous* than due to feeding by *S. montela* (Figure 4). Indeed, food shortage may have been a major factor responsible for the decreased *A. alcinous* survival due to increased conspecific density because no food remained (food depletion) at the end of the experiment in the single‐species treatment of *A. alcinous* when larval density was high (final food remaining: *S. montela* density was at a minimum (=0), *A. alcinous* density ≥8, Figure 4b). Therefore, the conspecific density effect on *A. alcinous* survival was mainly due to food shortage. In contrast, increasing *A. alcinous* density did not influence the survival or development time of *S. montela*, suggesting that there was little effect of food shortage on *S. montela* larvae. This is consistent with our finding of a lower food demand of *S. montela* than of *A. alcinous* (Table 1), although the causal relationships between their food demands and vulnerability to food shortage are unclear. One explanation may be that food demand is related to starvation tolerance. While larger species generally show higher starvation resistance than smaller species (Gergs & Jager, 2014), there is evidence that several factors can generate exceptions (Kirk, 1997; Stockhoff, 1991). Thus, it is possible that *S. montela* (smaller) has higher starvation resistance than *A. alcinous* (larger). If so, a reduction in food amount would more seriously affect *A. alcinous* than *S. montela*. As such, starvation tolerance may be a key point in understanding the mechanisms underlying the effects of food demand on vulnerability to food shortages.

Negative density effects on behavior, survival, and reproduction have been reported in other lepidopterans in the field (Dempster, 1983; Gibbs et al., 2004; Tammaru et al., 2000). However, few studies have compared density‐dependent responses between different species. Ammunét et al. (2010) showed that density effects differed between two geometrid moths; larvae of a larger moth, *Epirrita autumnata*, and a smaller moth, *Operophtera brumata*, were negatively affected by both con‐ and heterospecific larval densities, and the former experienced greater negative impacts on pupal mass than the latter. Because larger herbivores generally require more food for development than smaller herbivores (Brown & Maurer, 1986; Gaston & Lawton, 1988), *E. autumnata* may have experienced greater negative density effects than *O. brumata* because of stronger food limitation in *E. autumnata* given its greater food demand, which is consistent with the results of our study.

### 4.2 Mechanisms underlying heterospecific density effects

In contrast to *A. alcinous*, feeding by *S. montela* results in a slight reduction in plant quantity (Figure 4a, *A. alcinous* density = minimum), which is unlikely to cause food shortages. This is probably due to the difference in per capita consumption between these species. If the density effects on larval survival and development occurred only due to food shortage, the density effect of *S. montela* on *A. alcinous* was expected to be weaker than that of *A. alcinous*. Contrary to this expectation, the strength of the density effects on *A. alcinous* survival was not different between *A. alcinous* and *S. montela*. Moreover, in the treatments with mixed species, the final food remaining even increased with increasing *S. montela* density (Figure 4a, *A. alcinous* density = mean). These results imply that the negative density effect of *S. montela* on *A. alcinous* survival was unlikely to have occurred due to direct food shortage.

There are at least two possible mechanisms for the negative density effects of *S. montela* on *A. alcinous*: interference by *S. montela* and decreases in plant quality (e.g., chemical and physical defense and/or nutritional status) induced by *S. montela* (Denno et al., 1995; Kaplan & Denno, 2007; Ohgushi, 2005). Since the probability of both species being present on the same plant was low and we did not observe any direct offensive or interference behavior of *S. montela* larvae against *A. alcinous* larvae, a deterioration in plant quality caused by *S. montela* herbivory is more likely to have occurred. This is supported by the fact that *S. montela* density negatively affected the development of *A. alcinous* (Figure 3b). Since low plant quality often results in prolonged larval development time (Miller & Feeny, 1983, 1989), *S. montela* herbivory may reduce plant quality by increasing chemical/physical defense and/or decreasing nutritional status, leading not only to lower survival but also to prolonged development time in *A. alcinous*.

Because we did not examine the changes in plant quality following herbivory, how plant quality influences the effects of *S. montela* herbivory on *A. alcinous* is unclear. However, the specific secondary metabolite AAI did not affect the leaf consumption or growth of these butterflies, suggesting that this compound is unlikely to affect interactions between the two butterflies. This is not surprising, as these butterfly species are specialists, and a specific chemical defense may not be effective against them (Ali & Agrawal, 2012; Cornell & Hawkins, 2003). Therefore, plant attributes other than AAI, such as nonspecific defensive chemicals and/or nutritional status, may have been responsible for the decrease in plant quality induced by *S. montela* herbivory.

Note that the high *S. montela* mortality (more than 50% in all treatments) in the cage experiment compromised any con‐ and heterospecific density effects on *S. montela*. Although the mechanisms underlying such high larval mortality are not clear, one possibility is that larval dispersal may have resulted in *S. montela* mortality. In the field, larvae of *S. montela* and *A. alcinous* often move between plants (Inoue & Kon, 2006; Sakuratani et al., 2003), and the negative impact of larval dispersal may be greater in *S. montela* (smaller) than in *A. alcinous* (larger). In general, the cost of dispersal may be greater for smaller larvae than for larger larvae because smaller larvae take a longer time to find a new plant and are therefore more likely to die from starvation or ground predation before reaching a new plant (Rausher, 1979, 1981). Furthermore, our preliminary bioassay showed that neonate *S. montela* larvae rarely fed on mature leaves, whereas *A. alcinous* larvae can feed on young and mature leaves. If edible leaves for younger *S. montela* larvae were limited in the cage experiment, this may have promoted dispersal of younger *S. montela* larvae to seek other plants, thereby increasing mortality.

### 4.3 Causes of the asymmetry in the interspecific interaction between *S. montela* and *A. alcinous*

It has been suggested that interspecific interactions between herbivorous insects via shared host plants are often asymmetric, which challenges the traditional idea that competition is symmetric (Lawton & Hassell, 1981; Strong et al., 1984). Consistent with previous research, this study revealed an asymmetric interspecific interaction between the two specialist butterflies, where *S. montela* larvae negatively affected the larval performance of *A. alcinous*, but the reverse was not true. The differences in food demand as well as herbivore‐induced changes in plant quality may have played an important role in generating this asymmetric interaction. On the one hand, the reduction in food quantity caused by *A. alcinous* feeding did not influence *S. montela* larval performance, at least partly because of the lower food demand of *S. montela* larvae, which may be related to species‐specific starvation tolerance. On the other hand, feeding by *S. montela* may have deteriorated plant quality, leading to negative effects on *A. alcinous* larval performance. Recently, several reviews have argued that plant quality‐mediated indirect effects are responsible for asymmetric interactions between herbivorous insects (Denno & Kaplan, 2007; Kaplan & Denno, 2007). In this context, our study suggests that plant quantity also contributes to asymmetric interactions at least partially, if not equally to plant quality.

Although incorporating plant quantity into the understanding of plant‐mediated indirect interactions among herbivores has been suggested (Anderson et al., 2009; Morris, 1997), our knowledge of whether and how plant quantity affects plant‐mediated interactions is still limited. In this context, focusing on the food demands of herbivores would provide insights into how species‐specific traits of herbivores determine plant quantity‐mediated interactions. Although the key trait changes in *A. debilis* induced by *S. montela* feeding remain unclear, some characteristics of plant quality other than AAI, such as nutrient content, other secondary compounds, or physical traits, may be important. Future studies are needed to evaluate the responses of herbivores to changes in plant quantity, as well as quality, for a more exact understanding of plant‐mediated indirect interactions among herbivores.

## AUTHOR CONTRIBUTIONS

**Koya Hashimoto:** Conceptualization (lead); data curation (lead); formal analysis (lead); funding acquisition (supporting); investigation (lead); methodology (lead); project administration (lead); validation (equal); visualization (lead); writing – original draft (lead); writing – review and editing (lead). **Takayuki Ohgushi:** Conceptualization (supporting); funding acquisition (equal); methodology (supporting); project administration (supporting); resources (lead); supervision (lead); validation (equal); visualization (supporting); writing – original draft (supporting); writing – review and editing (supporting).

## CONFLICT OF INTEREST STATEMENT

The authors declare no conflicts of interest.

## Supporting information


Data S1.

## Data Availability

Data available from the Figshare Repository: https://doi.org/10.6084/m9.figshare.23170898.
