# Supplementary material for: Asymmetric interactions between two butterfly species mediated by food demand
Source: Ecol Evol. 2023 Jun 8;13(6):e10164. doi: 10.1002/ece3.10164 (PMC10249040; doi:10.1002/ece3.10164)
Supplement: Supplementary file 1 — Data S1. [file ECE3-13-e10164-s001.pdf]

Supporting information.

Title

Asymmetric interactions between two butterfly species mediated by food demand

Authors

Koya Hashimoto* and Takayuki Ohgushi

*: author for correspondence: atrophaneura4@gmail.com

Ecology and Evolution

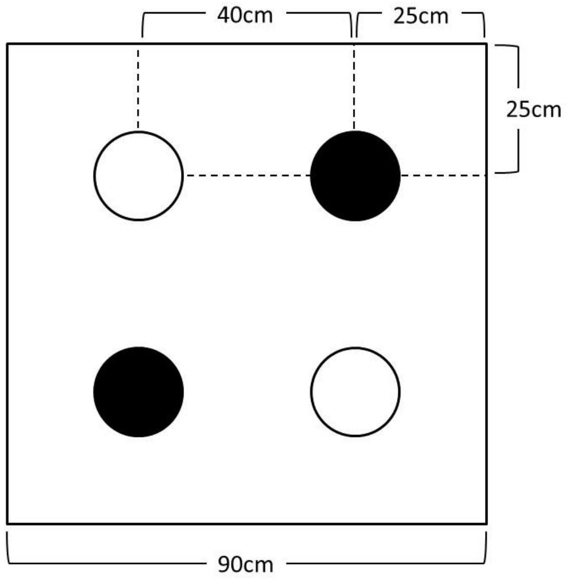

Fig. S1. Spatial arrangement of experimental plants in a cage. The four circles represent the pots in which the plants were germinated (one plant per pot). Neonate larvae of *S. montela* or *A. alcinous* were placed on either of the plants shown as black circles.

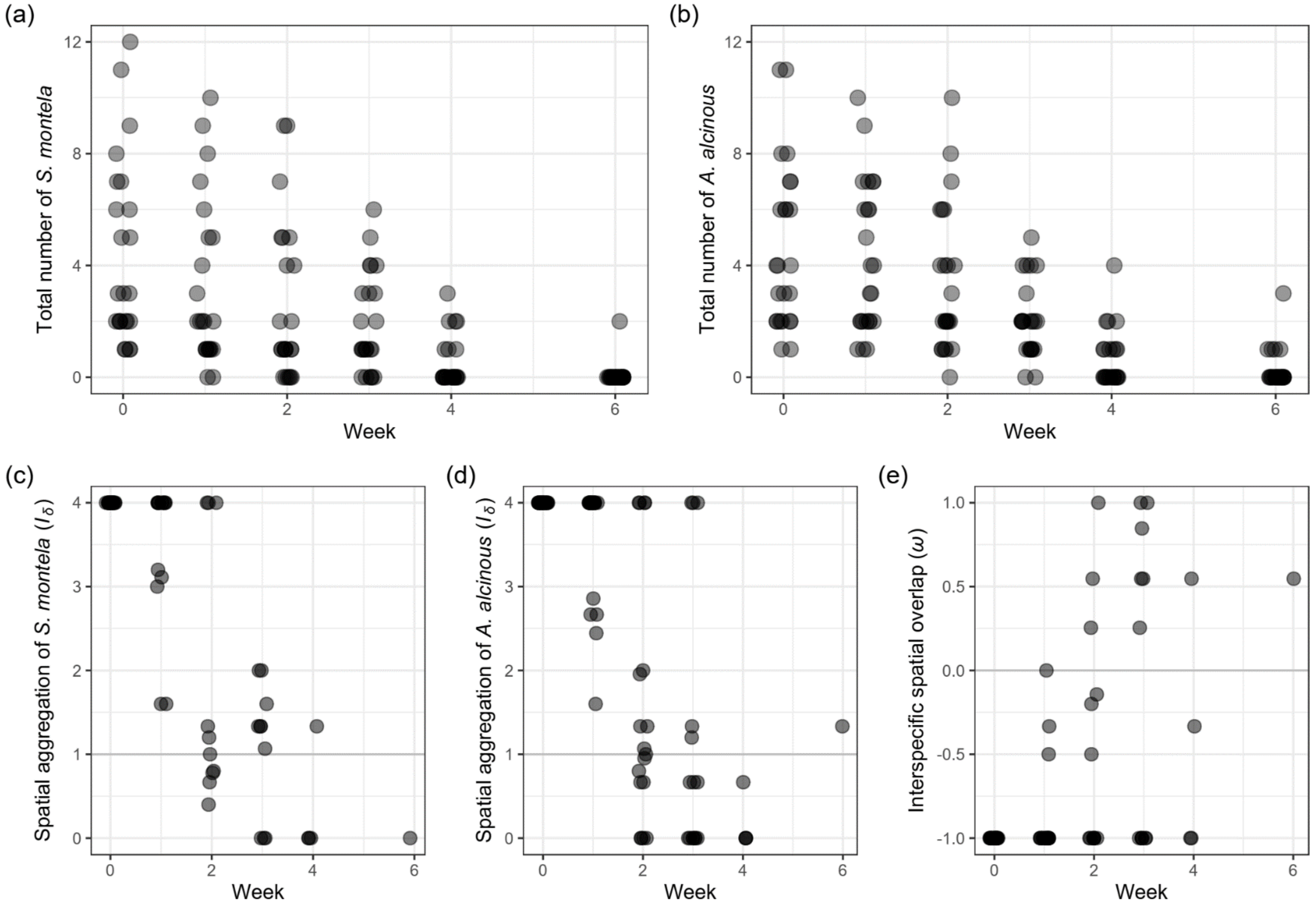

Fig. S2. (a-b) Temporal changes in the total number of (a) *S. montela* and (b) *A. alcinous* larvae in each cage. (c-e) Temporal changes in the spatial distribution of the two butterfly larvae on the experimental plants in each cage. Spatial aggregation of (c) *S. montela* and (d) *A. alcinous* and (c) interspecific spatial overlap between the two species are shown. Spatial aggregation was expressed by Morisita's I_δ index. $1 < I_\delta < +\infty$, aggregated distribution; $I_\delta = 1$, random distribution; $0 \leq I_\delta < 1$, dispersed distribution. Spatial overlap was given by Iwao's ω index. $-1 \leq \omega < 0$, segregated distribution; $\omega = 0$, random distribution; $0 < \omega \leq 1$, overlapping distribution. The data were collected 1 day and 7, 15, 21, 29, and 42 days after the experiment started.

Table S1. Temporal changes in the number of plants visited at least once by (a) *S. montela*, (b) *A. alcinous*, and (c) either *S. montela* or *A. alcinous* in each cage. The number in each cell indicates the number of cages. The data were collected 1, 7, 15, 21, 29, and 42 days after the experiment started. Note that in 20 out of 30 cages, all (four) plants were visited at least once by *S. montela* and/or *A. alcinous* larvae 6 weeks after the experiment started.

(a) *S. montela*

	1 plant	2 plants	3 plants	4 plants
0 weeks	24	0	0	0
1 week	17	6	1	0
2 weeks	11	6	4	3
3 weeks	9	4	7	4
4 weeks	9	3	7	5
6 weeks	9	3	6	6

(b) *A. alcinous*

	1 plant	2 plants	3 plants	4 plants
0 weeks	24	0	0	0
1 week	18	6	0	0
2 weeks	5	9	7	3
3 weeks	2	9	5	8
4 weeks	2	7	6	9
6 weeks	2	6	6	10

(c) Either *S. montela* or *A. alcinous*

	1 plant	2 plants	3 plants	4 plants
0 weeks	12	18	0	0
1 week	7	18	5	0
2 weeks	2	5	13	10
3 weeks	2	1	9	18
4 weeks	2	1	8	19
6 weeks	2	1	7	20

Table S2. The number of plants visited at least once by either *S. montela* or *A. alcinous* larvae in each cage 6 weeks after the experiment started, classified by initial total density treatments. The number in each cell indicates the number of cages. Note that in the 12 larvae treatment, in all (ten) cages, all (four) plants were visited at least once by *S. montela* and/or *A. alcinous*.

	1 plant	2 plants	3 plants	4 plants
4 larvae	2	1	4	3
8 larvae	0	0	3	7
12 larvae	0	0	0	10
